# Innovative Cell and Platelet Rich Plasma Therapies for Diabetic Foot Ulcer Treatment: The Allogeneic Approach

**DOI:** 10.3389/fbioe.2022.869408

**Published:** 2022-05-02

**Authors:** Maddalena Mastrogiacomo, Marta Nardini, Maria Chiara Collina, Cristiana Di Campli, Gilberto Filaci, Ranieri Cancedda, Teresa Odorisio

**Affiliations:** ^1^ Dipartimento di Medicina Interna e Specialità Mediche (DIMI), Università degli Studi di Genova, Genova, Italy; ^2^ Unità Operativa Semplice Piede Diabetico e Ulcere Cutanee, IDI-IRCCS, Roma, Italy; ^3^ IRCCS Ospedale Policlinico San Martino, Genova, Italy; ^4^ Emeritus Professor, Università degli Studi di Genova, Genova, Italy; ^5^ Laboratorio di Biologia Molecolare e Cellulare, IDI-IRCCS, Roma, Italy

**Keywords:** diabetic foot ulcers, PRP, cell therapy, advanced therapy, allogeneic preparations

## Abstract

Cutaneous chronic wounds are a major global health burden in continuous growth, because of population aging and the higher incidence of chronic diseases, such as diabetes. Different treatments have been proposed: biological, surgical, and physical. However, most of these treatments are palliative and none of them can be considered fully satisfactory. During a spontaneous wound healing, endogenous regeneration mechanisms and resident cell activity are triggered by the released platelet content. Activated stem and progenitor cells are key factors for ulcer healing, and they can be either recruited to the wound site from the tissue itself (resident cells) or from elsewhere. Transplant of skin substitutes, and of stem cells derived from tissues such as bone marrow or adipose tissue, together with platelet-rich plasma (PRP) treatments have been proposed as therapeutic options, and they represent the today most promising tools to promote ulcer healing in diabetes. Although stem cells can directly participate to skin repair, they primarily contribute to the tissue remodeling by releasing biomolecules and microvesicles able to stimulate the endogenous regeneration mechanisms. Stem cells and PRP can be obtained from patients as autologous preparations. However, in the diabetic condition, poor cell number, reduced cell activity or impaired PRP efficacy may limit their use. Administration of allogeneic preparations from healthy and/or younger donors is regarded with increasing interest to overcome such limitation. This review summarizes the results obtained when these innovative treatments were adopted in preclinical animal models of diabetes and in diabetic patients, with a focus on allogeneic preparations.

## 1 Introduction

Skin ulcers are open sores often accompanied by the sloughing-off of inflamed tissue. A slow-healing ulcer of the leg (usually the lower leg) is typically associated with complications of poor blood circulation, such as varicose veins, deep venous insufficiency, arterial and peripheral vascular diseases. Other causes of leg ulceration include trauma, bacterial and/or mycotic infections, and neuropathy related to diabetic disease. Pressure ulcers (bed sores) are a very common complication in elderly hypomobile patients. Cutaneous chronic wounds represent a major global health burden in continuous growth since this pathology is closely linked to the higher incidence in the aging population of chronic diseases, including diabetes. Women experience more pain and have a worse quality of life than men (Bartley and Fillingim, 2013; Rovner et al., 2017). There is a direct correlation between pain and quality of life, which is worse for ulcers with a longer duration and a larger area (Guarnera et al., 2007).

The prevalence of skin ulcers and the cost of treatments are very high. In industrialized countries, it has been estimated that 1%–2% of the population will experience a chronic wound during their lifetime (Sen et al., 2009). Lower limb ulcers represent a major clinical problem particularly for diabetic patients (Nabuurs-Franssen et al., 2005). In Europe 5%–7% of the population suffers from diabetes and this is expected to increase significantly during the next 20 years, especially in the elderly. It is estimated that up to 25% of all diabetics will develop an ulcer (Wu et al., 2007; Armstrong et al., 2017).

Chronic ulcers are difficult to heal because of the diminished blood flow interfering with the healing process. Patient care is concerned with preventing a superimposed infection in the ulcer, increasing blood flow in the deeper veins, and decreasing pressure within the superficial veins. Different treatments have been proposed: biological, surgical, and physical. However, these treatments are mostly palliative and none of them can be considered fully satisfactory. More recently, treatments with allogeneic stem/progenitor cells or platelet-rich plasma (PRP) have been proposed as alternative therapeutic options. This review summarizes the results obtained when these innovative treatments were adopted in diabetic ulcer patients.

## 2 Healing Defects in Diabetes

Skin repairs through a complex process that has been conventionally divided into four sequential and partly overlapping phases: prompt blood hemostasis is followed by inflammation, active cell proliferation, and long-lasting tissue remodeling (Shaw and Martin, 2009). Resident cell populations, together with cells recruited from the bloodstream, contribute to wound healing through a continuous molecular crosstalk as well as *via* interactions with the extracellular matrix (ECM). Platelets entrapped in the provisional fibrin clot release cytokines, primarily the stromal-derived factor-1 (SDF-1, also named CXCL12), and growth factors that promote immune cell recruitment and resident cell activation. Hepatocyte growth factor (HGF), platelet-derived growth factor (PDGF), transforming growth factor-*β*1 (TGF-*β*1) and epidermal growth factor (EGF) are among growth factors released by platelets (Barrientos et al., 2008). Neutrophils, monocytes, and lymphocytes in sequence invade the wound bed. Together with resident immune cells, they trigger responses necessary for recovering tissue sterility and for promoting skin regeneration. They increase reactive oxygen species (ROS) production, and release cytokines, growth factors and antimicrobial peptides (Barrientos et al., 2008; Brazil et al., 2019). Monocytes differentiate into M1 macrophages releasing several proinflammatory mediators. This reach milieu activates resident cell types that start proliferating and migrating. Pro-angiogenic growth factors released by anti-inflammatory M2 macrophages and by migrating keratinocytes (Brown et al., 1992) promote neoangiogenesis that sustains the high metabolic demand of the regenerative phase. The newformed skin is far from being a functional tissue, as it manifests hypertrophic epidermis, irregularly deposited and tick matrix, high cellularity, and excessive blood vessel number. Apoptotic cell removal and matrix reorganization restore skin homeostasis in the remodeling phase. The wound healing process often ends up with a scar that lacks dermal annexes and manifests reduced tensile strength. Dysregulation of the events guaranteeing wound repair may result in either loss of healing with chronic ulcer formation or excessive healing with aberrant scar development (Eming et al., 2014).

The difficult healing and the evolvement of diabetic wounds to chronic ulcers is multifactorial: wound infection, deregulated inflammatory response, abnormally increased oxidative stress, impaired angiogenesis, cell senescence and aberrant extracellular matrix deposition play major roles (Falanga, 2005). These pathogenetic mechanisms are in large part common to venous and arterial ulcers, in which insufficient oxygen and nutrient supply underlie the impaired healing response. As for diabetes, increased glucose levels elicit a specific pathogenetic response due to molecular glycation. Advanced-glycation end products (AGE) interact with their receptors (RAGE) at the surface of different cell types and, through activation of the NF-kB transcription factor, promote ROS overproduction and release of inflammatory mediators (Wautier et al., 1996; Goldin et al., 2006). Glycation is responsible for vasculopathy and peripheral neuropathy, it affects molecular function, increases ECM deposition and crosslinking and, in general, it impairs the activity of the different cell types involved in the healing process.

It is widely recognized that in diabetic wounds cell proliferation, migration, differentiation, and ability to release growth factors are impaired (Spravchikov et al., 2001; Lerman et al., 2003; Thangarajah et al., 2009; Cianfarani et al., 2013; Gallagher et al., 2015; Berlanga-Acosta et al., 2020; Sawaya et al., 2020). The number of recruited circulating cells is reduced due to decreased release of and/or response to chemotactic factors (Tchaikovski et al., 2009; Sawaya et al., 2020). Angiogenesis is profoundly hampered for nitric oxide (NO) deficit, reduced release of angiogenic factors, decreased recruitment and differentiation of hematopoietic and endothelial precursors (Tepper et al., 2010; Kolluru et al., 2012). Cells of the immune system manifest uncontrolled activity in diabetic wounds: initial inflammatory response is impaired facilitating colonization by pathogens, while persistent presence of inflammatory cells and increased cytokine and protease production strongly contribute to healing failure at later stages. Macrophages are major actors in the wound healing process, as well as in the abnormally prolonged inflammatory phase of diabetic wounds when they fail to polarize from the M1 pro-inflammatory phenotype into the M2 anti-inflammatory/regenerative one (Mirza and Koh, 2011; Mirza et al., 2014).

Altered cell behavior in diabetes also depends on oxidative stress- and inflammation-driven epigenetic changes which are maintained after reversal to a normoglycemic condition (Cencioni et al., 2014). This phenomenon, named as “hyperglycemic memory” or “metabolic memory,” likely plays a role in the high rate of recurrence in diabetic ulcers. Changes in global DNA methylation and deregulation in non-coding RNA expression have been described in diabetic skin and wounds (den Dekker et al., 2019; Ozdemir and Feinberg, 2019). When global microRNA expression was analyzed in non-lesional skin of type 1 and type 2 diabetic mice a general transcriptional impairment affecting the expression of microRNA precursors and biogenesis gene levels was found (Baldini et al., 2020). This data confirms the complexity of molecular defects in diabetes. Based on this evidence, modification of altered epigenetic marks have been suggested as therapeutic strategies against diabetic complications (Spallotta et al., 2013; den Dekker et al., 2019).

## 3 Innovative Therapies for Diabetic Foot Ulcers

Chronic skin ulcers are the most common diabetic complication causing pain and poor quality of life for patients. They frequently develop at foot, being referred as diabetic foot ulcers (DFU). Diabetic neuropathy (damage to the foot’s sensory nerves) results in foot deformities and/or ulcers that increase the chance of lower-extremity amputations when no treated. When concomitant peripheral arteriopathy occurs, the risk of DFU development strongly increases (Volmer-Thole and Lobmann, 2016).

A global prevalence of more than 6% was estimated for DFU with consequences on clinical and social costs (Zhang et al., 2017; Raghav et al., 2018). Some amputation is needed in approximately 20% of diabetic patients with an ulcer, and the risk of death increases up to 70% after amputation (Armstrong et al., 2017). Finally, ulcers recur in around 40% of patients within 1 year of remission, and this percentage increases over time, being over 60% after 5 years (Armstrong et al., 2017).

**FIGURE 1:**
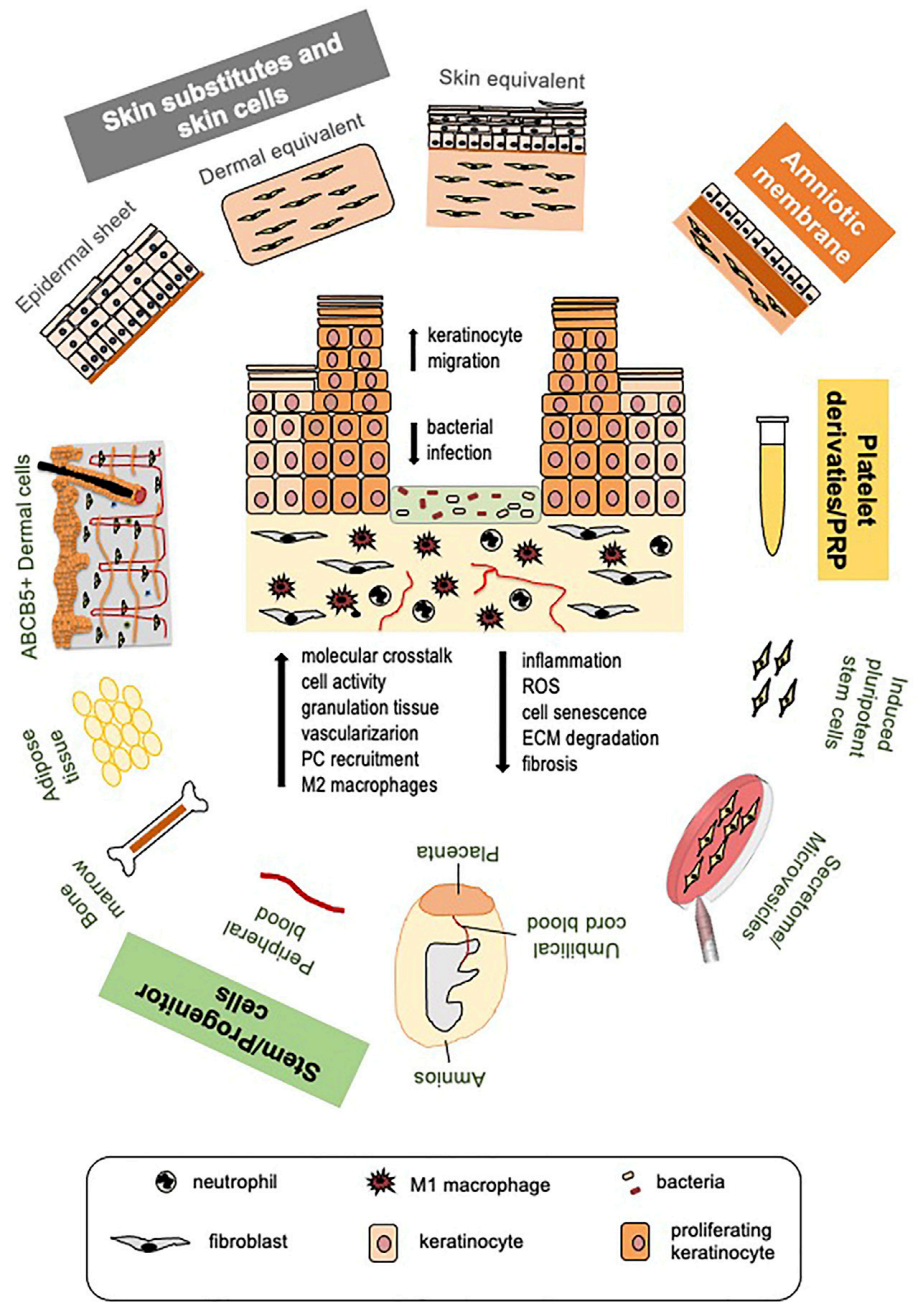
Cell and platelet-derived tools for diabetic ulcer therapy. Different cell or platelet-derived tools with the beneficial effects they elicit on diabetic wound healing (a diabetic ulcer schematic is depicted in the center). Skin cells and skin substitutes, stem cells of different origin and their secretome, platelet derivatives and platelet-rich plasma (PRP) are all potential tools to promote healing of diabetic ulcers. Induced pluripotent stem cells (iPSCs) derived from fibroblasts are promising therapeutic tools for improving autologous and possibly allogeneic cell therapy, not yet tested at clinical level. Therapeutic tools are applied after routine removal of necrotic tissue and antiseptic treatments. Beneficial effects of all approaches are largely due to cell ability to release great amount of growth factors, cytokines and chemokines, and other biomolecules; for this reason, MSC secretome and released microvesicles are being tested as cell-free, safer therapeutic approaches. These therapies increase the molecular crosstalk, promote cell function and, likely, circulating precursor cell recruitment. Re-epithelialization is stimulated due to enhanced keratinocyte migration; granulation tissue formation and vascularization are improved. Mesenchymal stem cells (MSCs), the amniotic membrane and PRP specifically manifest immunomodulatory and immunosuppressive properties leading to reduced wound inflammation and scar tissue formation, allowing allogeneic cell and PRP use without major adverse effects. (PC, precursors cells; ROS, reactive oxygen species; ECM, extracellular matrix).

Standard DFU management comprises the removal of necrotic tissue (debridement), interventions on the infection, application of dressings to protect the wound and maintain a moist environment necessary for promoting cell activity, offloading, and strict glycemic control (Everett and Mathioudakis, 2018). Surgical intervention for correcting vascular insufficiency can be considered in ischemic ulcers. In the absence of an active healing response, advanced and innovative therapies are considered. Adoption of skin substitutes, amniotic membrane allografts, stem cell-based therapies, including conditioned medium and released microvesicles, or platelet-rich plasma (PRP) treatments represent the most promising tools to promote healing in diabetes (Figure 1).

### 3.1 Skin Substitutes and Skin Cells

Skin grafts are the most obvious therapeutic approach for replacing the lost tissue of skin ulcers. Cadaveric cryopreserved skin allografts can be used to stimulate healing, particularly for deep wounds with exposed bones and tendons (Snyder and Simonson, 1999). Besides creating a temporary barrier, they act by stimulating re-epithelialization and granulation tissue formation; however, a retrospective comparative analysis on the effectiveness of cadaveric skin allograft against bioengineered living cell constructs revealed that living cells manifest better performance (Treadwell et al., 2018).

Since the pioneering works of Gallico et al. (1984) and Boyce et al. (1995), keratinocytes and/or fibroblasts have been topically applied as epidermal or dermal substitutes or as bilayer engineered constructs of both stratified keratinocytes and matrix-embedded fibroblasts. Engineered bilayer substitutes better reproduce skin structural and functional properties, as the crosstalk between keratinocytes and fibroblasts reinforces the activity of both cell types (Dellambra et al., 2019). Three dimensional bioprinting technology is often used for skin equivalent construction (Tan et al., 2020). 3D-bioprinted skin equivalents may contain endothelial cells and pericytes forming vascular structures that interconnect with host vasculature. These complex constructs were able to promote stable engraftment in a preclinical setting (Baltazar et al., 2020).

Autologous keratinocytes and dermal fibroblasts were found to promote diabetic ulcer healing in few clinical studies. In a small group of patients with deep diabetic ulcers (Wagner degree 3), autologous fibroblast grafts induced complete re-epithelialization, with no recurrence for at least 2 years (Cavallini, 2007). A very good rate of healing was also obtained in a pilot study with keratinocytes (Lobmann et al., 2003). A large observational retrospective trial with autologous fibroblasts and keratinocytes applied to wound with a semisynthetic scaffold (HYAFF 11 system) showed a good rate of complete healing, with low recurrence and excellent safety profile (Uccioli, 2003). This study lacked a control group; when the same system was used in a controlled, randomized clinical study on type 1 and type 2 diabetic ulcers, a significant improvement in healing was observed in dorsal but not plantar ulcers (Caravaggi et al., 2003). A recent meta-analysis on the effectiveness of split thickness skin grafts for diabetic foot and leg ulcers reported a global healing rate of 85.5% after a median time of only 5.35 weeks, with a recurrence rate of 4.2% after 2 years (Yammine and Assi, 2019). These numbers are far better as compared to those obtained with standard treatments.

Allogeneic skin cells from non-diabetic donors were also used to promote healing of chronic DFU without major safety problems being encountered (Table 1). Keratinocytes and fibroblasts can be isolated from neonatal foreskin. These cells not only manifest an active phenotype in terms of proliferation, migration and paracrine activity, but also likely promote a scarless healing due to intrinsic properties (Moore et al., 2018). A preliminary study suggested that singularly administered keratinocyte sheets and dermal substitutes are equally effective in promoting healing of diabetic ulcers (Harvima et al., 1999).

**TABLE 1:** Clinical studies on administration of allogeneic cell tools to diabetic ulcers: MSCs, mesenchymal stem cells; Ad-MSCs, adipose tissue-derived MSCs; Pl-MSCs, placenta-derived MSCs; UCB-MSCs, umbilical cord blood-derived MSCs; HSCs, hematopoietic stem cells; ASC, adipose stem cells.

Cell type	Type of study	Administration	N. patients	Effects	References
Keratinocyte epithelium or dermal fibroblasts in gelatin sponge	Case report	Weekly until healing	21, type I and II diabetes (26 ulcers)	All ulcers healed	Harvima et al. (1999)
Primary keratinocyte in a hyaluronic acid scaffold	Clinical case series	Once. Patients observed until 70 days	11, type II diabetes (16 ulcers)	Mean wound area reduction 70%. One ulcer with local severe infection	Marchesi et al. (2014)
Primary foreskin keratinocyte sheet (Kaloderm, Tego Science)	Prospective observational	Weekly (or 2–3 times/week if necessary) until 12 weeks	71, type I and type II diabetes	64.8% of complete healing. No adverse effects	Hwang et al. (2019)
Primary adult keratinocytes attached onto microcarriers	Case-control (double arm)	Every 3 days until healing	40, randomized into two groups of 20	Wound area reduction 92% (treated) vs 32% (controls), at 30 days. Improved wound score	Bayram et al. (2005)
Primary foreskin keratinocyte sheet on vaseline gauze	Case-control (double arm)	Weekly for 11 weeks	59, type I or type II diabetes; 27 cases and 32 controls	Complete healing in 100% of treated wounds and 69% of controls. No adverse effects	You et al. (2012)
Primary fresh dermal fibroblasts from teenagers embedded in fibrin	Case-control (double arm)	Single application	55, type I and type II diabetes; 37 cases and 18 controls	Complete healing in 83.8% of cases and 50.0% of controls at 8 weeks. No adverse effects	Han et al. (2009)
Dermagraft	Randomized, multicenter	Weekly, up to 7 treatments	245, type I or type II diabetes; 130 cases, 115 controls	Complete healing in 30% of treated patients and 18.3% of controls by week 12. Adverse events similar in the two groups	Marston et al. (2003)
Bilayered allografts (Graftskin)	Randomized, multicenter	Weekly, up to 4 weeks (5 times)	208, type I or type II diabetes, 112 treated and 96 controls	Complete healing in 56% of treated patients and in 38% of controls. Adverse effects similar in the two groups	Veves et al. (2001)
Foreskin fibroblasts + UCB-MSCs + HSCs	Pilot study	Single application, follow-up for 12 weeks	4 diabetics with severe PAD	Wound healing from 80 to 90%, reduced rest pains. No adverse effects	Viswanathan et al. (2013)
Pl-MSCs (Cenplacel)	Phase 1, dose-escalation, multicenter	Two applications (day 1 and 8), 24 months follow-up	15, type I or type II diabetes and PAD	7 patients had some degree of healing after 3 months (5 complete healing). ABI was improved in them. No severe adverse effects at all doses	Wu et al. (2017)
AD-MSCs (Allo-ASC-Sheet)	Phase 2, randomized, single-blind	Weekly, 12 weeks follow-up	39, type I and type II diabetes; 22 cases and 17 controls	Wound healing in 82% of treated and 53% if controls, at 12 weeks. Similar HLA levels in the two groups. No serious adverse events	Moon et al. (2019)
Allogeneic Ad-MSCs	Phase I/2, randomized single-blind	Single treatment, mean follow-up 48 months (26-50 months)	20, type II diabetes; 10 cases and 10 controls	Significant acceleration in wound closure in the treatment group	Uzun et al. (2021)

Several clinical studies proved the ability of keratinocytes from healthy donors to safely reactivate the healing response in DFU non-responding to conventional treatments. Epidermal substitutes were administered on a hyaluronic acid support (Marchesi et al., 2014; Marchesi et al., 2020), on vaseline gauze (You et al., 2012; Hwang et al., 2019), or loaded on microcarriers (Bayram et al., 2005).

Allogeneic dermal substitutes with normal fibroblasts plated in a spongy matrix of hyaluronic acid and atelo-collagen were used as dressings to promote granulation tissue formation prior to autologous skin grafting (Hasegawa et al., 2005). The allogeneic cryopreserved dermal substitute Dermagraft (Advanced BioHealing Inc., La Jolla, CA, United States), approved by the United States Food and Drug Administration (FDA) for DFU therapy, was tested in a multicenter randomized clinical trial on 245 patients with neuropathic foot ulcers (Marston et al., 2003). It improved the rate of wound closure at 12 weeks, with an incidence of adverse effects like conventional treatments. Despite the observed good results, cryopreserved substitutes manifest high cell death rate and reduced growth factor release compared with fresh preparations (Mansbridge et al., 1998). On the other hand, working with freshly prepared cells has obvious limitations, such as availability of skin cell donations and maintenance of cell culture standards. A fresh human allograft containing dermal fibroblasts from teenagers strongly promoted healing with no safety complications in a pilot study on diabetic chronic ulcers (Han et al., 2004). A subsequent case-control study using the same allograft confirmed the beneficial effect of this construct, with improved mean healing time and patient satisfaction (Han et al., 2009). No adverse effects were recorded.

Better performances were achieved with allogeneic implants containing both keratinocytes and fibroblasts. Apligraf (Organogenesis, Inc., Canton, MA, United States), approved by FDA for diabetic ulcer therapy, is a cryopreserved, bi-layered allograft, formed by foreskin-derived fibroblasts seeded within a bovine collagen I matrix overlaid by a stratified epithelium of neonatal keratinocytes (Zaulyanov and Kirsner, 2007). A pivotal, randomized multicenter prospective trial showed that Apligraf application to non-infected neuropathic diabetic ulcers significantly increases healing rate, reduces healing time, with no immunological reaction and with long-term reduction of osteomyelitis and lower-limb amputations (Veves et al., 2001).

### 3.2 Stem/Progenitor Cells of Different Tissue Origin

#### 3.2.1 Mesenchymal Stem Cells

Progenitor cells of mesodermal origin, also named mesenchymal stem cells (MSCs), reside in many tissues, including dermis and skin annexes (Hoogduijn et al., 2006; Hasebe et al., 2011; Ma et al., 2018) (Figure 1). It is still debated whether circulating mesodermal progenitors exist that could home to the wound site through a chemokine gradient (Rafii, 2000; Lo Sicco et al., 2018).

MSCs from different tissues share the expression of specific cell surface markers and lack those of hematopoietic and endothelial cells (Maleki et al., 2014; Camilleri et al., 2016). They manifest high self-renewal and the potential to differentiate into different cell types depending on tissue microenvironment (Pittenger et al., 2019). Although preclinical tracing experiments with labelled MSCs suggested that these cells may differentiate into keratinocytes, endothelial cells and pericytes (Sasaki et al., 2008; Nie et al., 2011), trans-differentiation minimally, if any, contributes to skin regeneration (Phinney and Prockop, 2007). MSC beneficial properties are largely due to paracrine activity and their ability to remodel the tissue through regulating resident cells by releasing growth factors, cytokines, microRNAs, etc. (Mansbridge et al., 1998; Brem et al., 2003; Lazic and Falanga, 2011).

A great number of studies in animal models of diabetes proved MSC efficacy in promoting skin repair (Cao et al., 2017). Stem cell administration reduces inflammation, apoptosis and scar formation, while increases cell proliferation and angiogenesis. Human studies and clinical trials privileged the use of autologous MSCs for safety clues. However, clinical translation gave variable results and was not as effective as hoped.

A critical issue with MSC therapeutic potential is the poor cell survival and engraftment (Baldari et al., 2017). To reinforce MSC function or to enable them to better cope with the hostile microenvironment, several approaches were used (Wiredu Ocansey et al., 2020) (for reviews, Bardali et al., 2017; Shojaei et al., 2018). Scaffolds made by fibrin (Stolzing et al., 2011), collagen (Assi et al., 2016), hydrogels (Wong et al., 2011; Dong et al., 2018) or acellular dermal matrix (Fu et al., 2019) were adopted for MSC delivery and to enhance their therapeutic potential. MSCs cultured in the presence of selective growth factors or engineered to overexpress them showed enhanced key healing functions, particularly angiogenesis and progenitor cell recruitment, in diabetic preclinical settings (Di Rocco et al., 2010; Li et al., 2013; Penna et al., 2013; Yang et al., 2013; Capilla-González et al., 2018; Dhoke et al., 2020; Srifa et al., 2020). Hypoxia pre-treatment also enhanced MSC survival in the diabetic wound environment by minimizing ROS accumulation and improving angiogenesis (Liu et al., 2015). In general, preconditioning MSCs by exposure to physical or environmental shocks promoted their survival (Baldari et al., 2017).

The first and most used autologous MSCs for promoting diabetic wound healing were bone marrow derived (BM-MSCs). BM-MSCs provided promising results in both preclinical and clinical studies (Falanga et al., 2007; Cao et al., 2017). However, bone marrow aspiration and *ex vivo* expansion represent limiting factors in their use. Adipose tissue-derived MSCs (AD-MSCs) manifest properties similar to BM-MSCs (Strioga et al., 2012), but, unlike the bone marrow, adipose tissue is easily accessible and abundant in the body. The therapeutic use of AD-MSCs for DFU treatment has been extensively investigated, at both preclinical and clinical level (Gadelkarim et al., 2018). Additional types of investigated MSCs were derived from fetal or extraembryonic tissues. Among them, umbilical cord blood (UCB) MSCs are easily collectable and manifested efficiency in promoting non-diabetic and diabetic wound healing by exerting multiple beneficial effects (Moon et al., 2017; Zhang et al., 2020). However, privacy concern, and high cost of cord blood preservation may represent limitations in their use (Cao et al., 2017). MSCs from the Wharton’s jelly tissue (WJ-MSCs) are emerging as the best umbilical cord-derived MSC population for regenerative medicine. Besides being easily accessible and numerous, WJ-MSCs show features of embryonic stem cells (ESCs) in terms of proliferation, multipotency and low immunogenicity, but do not form teratomas upon transplantation as ESCs do and there are not ethical limitations in their use (Liau et al., 2020). WJ-MSCs also proved to efficiently promote diabetic wound healing in preclinical studies (Jiao et al., 2021; Nilforoushzadeh et al., 2022). Other MSCs that got attention for their ability to promote diabetic wound healing are those obtained from the placenta (Pl-MSCs) or the amniotic fluid (AF-MSCs) (Kim et al., 2012; Kong et al., 2013; Lee et al., 2015; Wu et al., 2017) (Figure 1).

A small subpopulation of mesodermal skin cells manifesting MSC features has been recently identified (Riedl et al., 2021). These cells express the ATP-binding cassette subfamily B member 5 (ABCB5+ MSCs). They show strong immunomodulatory properties and better homing to skin wounds as compared to BM-MSCs (Schatton et al., 2015). A preclinical study on a mouse model of venous leg ulcer revealed that ABCB5+ MSCs favor healing by promoting macrophage polarization towards the M2 phenotype, and that this effect is mediated by the release of the interleukin-1 receptor antagonist (IL-1RA) (Vander Beken et al., 2019). Ex vivo-expanded autologous ABCB5+ MSCs were used in a clinical study with few patients affected by chronic venous ulcers (Kerstan et al., 2021). These cells facilitated healing and promoted pain relief with no adverse effects.

When considering the adoption of MSCs as therapeutic agents for DFU, one should be aware that preclinical data showed that the diabetic environment affects the beneficial activity of AD-MSCs and BM-MSCs in promoting wound healing. The number of MSCs is decreased in both type 1 and type 2 diabetes and their proliferative potential, paracrine activity, survival, and recruitment to the wound site are altered (Shin and Peterson, 2012; Cianfarani et al., 2013; Ribot et al., 2017). In a study on type 1 diabetic mice we demonstrated that the allogeneic stromal vascular fraction (SVF) from the adipose tissue of non-diabetic mice was more effective in promoting healing of diabetic wounds as compared to SVF from diabetic mice, in terms of granulation tissue formation, angiogenesis and macrophage recruitment (Cianfarani et al., 2013). Other preclinical studies also showed that the angiogenic response is strongly impaired when MSCs from diabetic animals are transplanted (El-Ftesi et al., 2009; Ribot et al., 2017). By using mouse models, Rennert et al. (2014) found that the AD-MSC niche is altered in diabetes and hypothesized that the reduced angiogenic response of diabetic AD-MSCs depends on the selective ablation of a subpopulation with putative strong angiogenic activity. These preclinical findings were confirmed in humans, where reduced amounts of circulating MSCs, growth factors and anti-oxidant molecules were found in the plasma of type 2 diabetic patients as compared to non-diabetic individuals (Nowak et al., 2014). A selective depletion of a proangiogenic CD271+ AD-MSC population was also found in diabetic individuals with cardiovascular diseases (Inoue et al., 2019), and human MSCs exposed to sera of type 2 diabetic patients revealed decreased survival and *in vitro* proangiogenic activity (Rezabakhsh et al., 2017).

**FIGURE 2:**
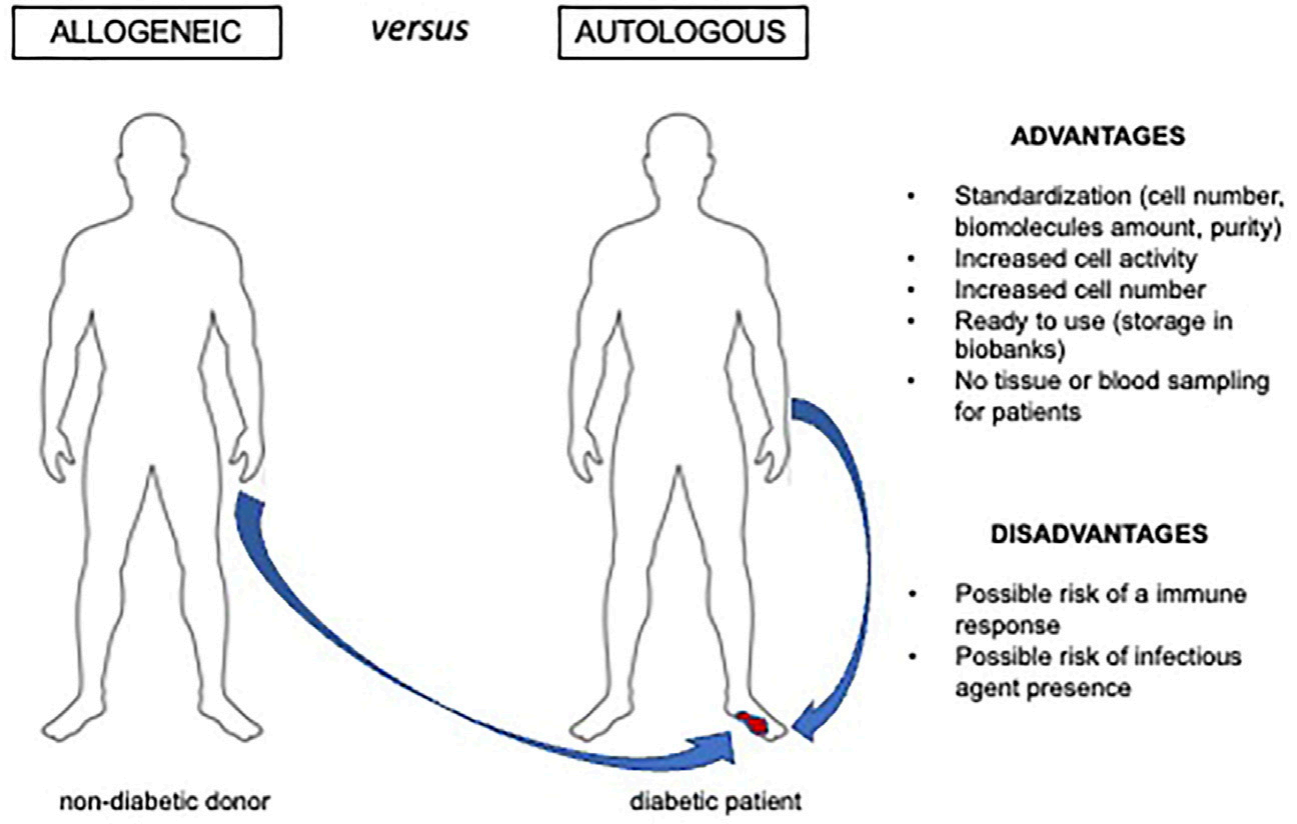
Advantages and disadvantages of allogeneic cell. therapy Allogeneic cells or cell derivatives can be an option for ulcer therapy when harvesting autologous preparations able to reactivate the healing process is not feasible. This is the case of diabetic and/or elderly individuals that manifest impaired cell number and activity.

Allogeneic MSCs from healthy donors could be a valid therapeutic option to overcome issues of reduced number and activity of diabetic MSCs (Figure 2). This approach is feasible as MSCs manifest immune tolerance. Indeed, MSCs express low level of human leukocyte antigen class I (HLA-I) (Wang et al., 2019) and inhibit inflammatory responses. Human MSCs cultured in the presence of different immune cell populations were shown to mitigate immune responses by reducing the release of proinflammatory cytokines (IFN-*γ*, TNF-*α*) and by increasing both anti-inflammatory cytokines (IL-4, IL-10), and prostaglandin E2 (PGE2) (Aggarwal and Pittenger, 2005). A recent meta-analysis on clinical studies employing allogeneic AD-MSCs for other pathologies confirmed that these cells are therapeutically effective with no severe adverse effects or evidence of immune response, even though 19%–34% of patients were found to develop antibodies against donors (Gentile et al., 2020). This could represent a potential risk for patients qualifying for a possible future organ transplant.

Following encouraging preclinical results, few preliminary clinical studies with allogeneic MSCs in chronic diabetic ulcers have been published (Table 1). A study on diabetic patients with amputation as only option for severe peripheral arterial disease (PAD), evaluated the combination of intramuscular administered allogeneic UCB-MSC and CD34+ hematopoietic stem cells with intralesional implantation of allogeneic foreskin fibroblasts (Viswanathan et al., 2013). After a 3 months therapy, all patients manifested an improvement in wound healing ranging from 80 to 90%, with healthy granulation tissue formation and no local, nor systemic complications. The ankle brachial index (ABI) was improved, and no limb amputation was necessary. Reduced rest pain was also reported, and no ulcer formation was observed after 6 months follow-up.

A phase 1 dose-escalation study aimed at evaluating safety of placental MSCs (Cenplacel) in patients with DFU and PAD showed no adverse effects at all doses tested (Wu et al., 2017). After 3 months, 7 out of 15 ulcers had healed and this effect was durable after 1 year. PAD was improved in patients whose ulcer healed. This therapeutic effect on peripheral vasculature was also reported in a preclinical study with the same therapeutic approach (Francki et al., 2016).

Twelve studies using allogeneic MSCs for diabetic ulcer treatment are presently recorded in the National Institutes of Health (NIH) clinical trial registry (www.clinicaltrials.gov). Of those, eight test the effect of a product containing allogeneic AD-MSCs from healthy donors in a hydrogel sheet (ALLO-ASC-Sheet). A randomized, single-blind, phase 2 clinical trial testing efficacy and safety of this commercial product versus standard therapy has been published (Moon et al., 2019). Despite the relatively small sample size, percentage of healed ulcers after 8 and 12 weeks was increased in the group treated with the ALLO-ASC-Sheet, and mean time to complete healing was significantly shorter in the treatment group. After 2 years follow-up, recurrence was minimal, and no adverse events were recorded. Increased elevation of anti-HLA antibodies was found in the treatment group, but no clear signs of rejections were detected. Among approved clinical trials, there is a phase 1-2a with *in vitro* expanded MSCs of dermal origin (ABCB5+-MSCs) in neuropathic DFU.

#### 3.2.2 Induced Pluripotent Stem Cells

Induced pluripotent stem cells (iPSCs) are obtained from differentiated adult cells following ectopic co-expression of genes coding for transcription factors conferring a pluripotent stem cell phenotype (Takahashi and Yamanaka, 2006). Under proper culture conditions, iPSCs can re-differentiate into different cell types, including skin cells (Itoh et al., 2013). This technology allows to obtain large number of rejuvenated, active cells.

Induced PSCs obtained from dermal fibroblasts and subsequently differentiated into smooth muscle cells showed an increased ability to release growth factors and to promote wound healing in diabetic mice when transplanted in a tridimensional scaffold (Gorecka et al., 2020). They improved cell proliferation, neovascularization, extracellular matrix deposition and M2 macrophage polarization.

Dermal fibroblasts from non-healing DFU were found to dedifferentiate to iPSCs as efficiently as fibroblast from non-lesional diabetic skin or from non-diabetic skin (Gerami-Naini et al., 2016). Kashpur et al. (2019) showed that iPSCs derived from non-healing DFU fibroblasts can re-differentiate to dermal fibroblasts that manifest a common gene expression signature with fibroblasts whose parental cells were non-diabetic. This genetic convergence with non-diabetic cells is likely due to epigenetic changes reversing the hyperglycemia-induced “metabolic memory.” Reprogrammed fibroblasts showed improved behavior in terms of composition of released extracellular matrix, and ability to migrate and to promote wound healing when grafted in a tridimensional matrix. These cells also manifested changes in microRNA expression, with an increase in microRNAs whose target genes contribute to the healing defects in diabetes and a decrease in those targeting genes that promote skin repair (Pastar et al., 2021).

Since iPSCs from dermal fibroblasts release a richer ECM compared to original fibroblasts, they have been used to develop tissue engineering scaffolds. These scaffolds enhance the activity of DFU fibroblasts that release higher amount of glucosaminoglycans, VEGF and anti-inflammatory cytokines, and reduce the secretion of proinflammatory cytokines (Santarella et al., 2020).

Based on these findings, iPSCs derived from dermal fibroblasts could be considered for a non-invasive cell therapy in DFU. However, strong regulatory constraints exist to their use, the methodology to obtain the cells is highly sophisticated, and, at present, iPSCs are produced only in very specialized laboratories.

### 3.3 Stem Cell Secretome and Released Microvesicles

Skin substitutes and stem/progenitor cells mainly act not by engrafting (Phillips et al., 2002; Griffiths et al., 2004), but rather by releasing molecules that stimulate the healing process, such as growth factors, cytokines and extracellular matrix proteins (Mansbridge et al., 1998; Brem et al., 2003; Lazic and Falanga, 2011). Moreover, a significant part of the positive effect of the treatments with stem and progenitor cells is most probably due to the release of microvesicles (MVs) carrying different biomolecules, including proteins, coding and non-coding RNAs and lipids (Tsiapalis and O’Driscoll, 2020). This brought scientists and physicians to evaluate the direct use of the cell-conditioned medium and/or of cell-released MVs to induce activation and reactivation of endogenous progenitors and differentiated cells (Ahangar et al., 2020; Bari et al., 2020; De Gregorio et al., 2020; Zhang et al., 2020).

In particular, MSC-secreted exosomes have been quite extensively tested at preclinical level as potential tools for cell-free therapy in cutaneous wound healing (Casado-Díaz et al., 2020). These nanovesicles manifest immunomodulatory and regenerative properties comparable to MSCs. At present, a phase 1 single-arm clinical trial with MSC secretome is registered as completed on the clinical trial registry (www.clinicaltrials.gov; NCT04134676). This study was aimed at testing the therapeutic potential of umbilical cord MSC on healing of chronic ulcers.

### 3.4 Amniotic Membrane Allografts

Among allogeneic approaches for diabetic foot ulcers, it has to be mentioned the amniotic membrane. This natural dressing is being used for decades to promote tissue regeneration and in skin repair as it releases factors promoting cell function, it is non-immunogenic, reduces pain, inflammation and fibrosis, among other properties (Schmiedova et al., 2021). Amniotic membranes have been widely tested in clinical settings and proved to significantly promote healing of recalcitrant DFU in combination with standard care approaches (Laurent et al., 2017). Commercially available amniotic membrane products are provided with or without chorion, as cryopreserved or dehydrated decellularized tools (Ilic et al., 2016). They can be also used as scaffold for other advanced therapeutic approaches, as assessed in preclinical and clinical studies (Hashemi et al., 2019; Zhou et al., 2019; Xiao et al., 2021).

### 3.5 Platelet Derivates, Platelet-Rich Plasma

Activated platelets release high amounts of growth factors and active molecules capable to trigger cell proliferation, matrix remodeling, angiogenesis and to modulate inflammation at the wound site (Backly et al., 2011; Greppi et al., 2011; Andia and Maffulli, 2013; Burnouf et al., 2013; Ulivi et al., 2014). Moreover, platelets also release microvesicles and exosomes (Aatonen et al., 2012). Platelet-rich plasma (PRP) or other platelet-derivatives can reactivate latent endogenous regeneration mechanisms.

*In vitro* experiments from our group showed an initial transient enhancement of the inflammatory response (NF-kB activation, COX-2 induction, and secretion of pro-inflammatory cytokines), followed by the establishment of an anti-inflammatory microenvironment due to prostaglandin E2 (PGE2) production (Ruggiu et al., 2013). At the same time, it was observed an upregulation of proliferative and survival pathways, such as ERKs and Akt, together with induction of Cyclin D1 and the phosphorylation of retinoblastoma protein leading to the re-entry in the cell cycle of quiescent cells. Moreover, PRP promoted recruitment at the wound site of neutrophils and macrophages, which, in turn, stimulated vascularization, and recruitment, activation, proliferation of mesenchymal and epithelial stem/progenitor cells, thus enhancing tissue repair (Pierce et al., 1989).

**FIGURE 3:**
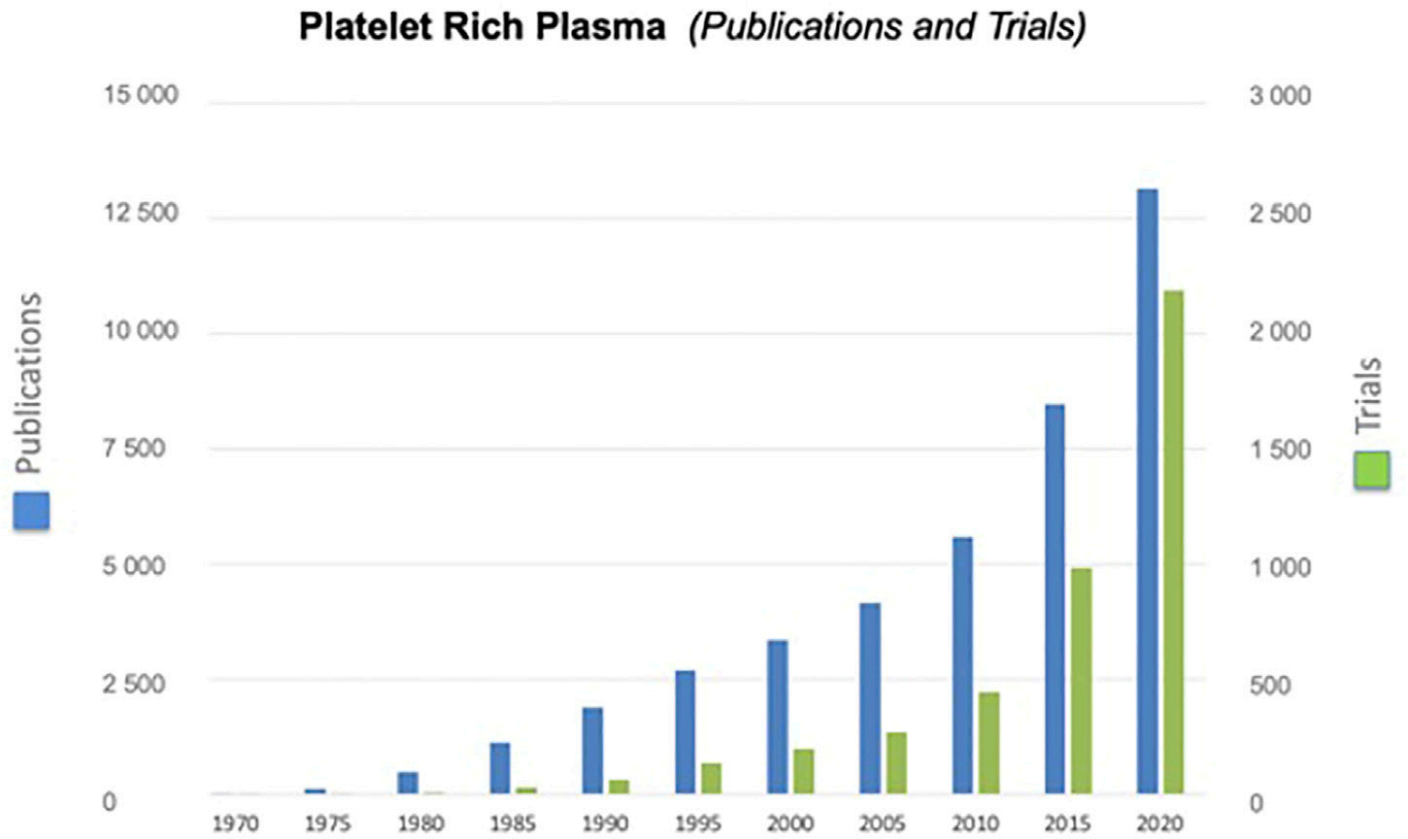
Exponential growth of publications and clinical trials having PRP as subject. Information was derived searching by “Platelet-Rich Plasma” the data base “PubMed” for publications and “Cochrane library (Title, Abstract, and Keywords)” for trials.

Platelet derivatives, primarily PRP, were considered in regenerative medicine to mimic the effects exerted by the platelet clot as wound healing trigger. Given its high availability as an autologous product and the easiness to prepare, PRP is being adopted in different medical fields, in particular in orthopedic and dermatology, for the treatment of chronic inflammatory diseases such as osteoarthritis and diabetic ulcers (Andia and Maffulli, 2013; Picard et al., 2015). Indeed, both the number of scientific publications and the number of clinical trials having PRP as subject is exponentially increasing (Figure 3). However, contradictory results are reported in the scientific literature on the outcome of treatments with autologous PRP. The main factor that could explain the different treatment outcomes is the variability of the preparations due to the lack of standardized procedures for PRP production. Moreover, as consequence of the blood recovery from a single donor, parameters, such as platelet concentration, and leukocyte, red blood cells and fibrin, change in different preparations and could be critical for the success of PRP treatment (Weibrich et al., 2002). Further variables arise from the different materials used for the recovery and processing of blood: vacuum blood-collection tubes, anticoagulant, blood collection bags together to time, speed, and number of centrifugations. In general, an important weakness of the clinical studies is the lack of defined parameters to assess the biological quality of the PRP such as the growth factor content and the testing of the product performance before its clinical use. Indeed, only a low percentage of trials included a quality control of the PRP preparations (Alsousou et al., 2009). It is also to note that several of the commercial devices approved by the FDA and other regulatory agencies for the preparation of autologous PRP were checked and approved for the safety, but not for the clinical efficacy of the obtained products (Harm and Fung, 2015).

Despite the above caveats, increasing evidence indicates that PRP is a powerful tool to enhance impaired wound healing. A recent Cochrane review tried to elucidate PRP efficacy on the treatment of recalcitrant ulcers. The meta-analysis is based on ten trials for a total of 442 participants. These studies were heterogeneous in terms of ulcer etiology: venous ulcers, diabetic ulcers, occlusive peripheral vascular disease, vasculitis and/or pressure ulcers. The median wound duration and size were similar at baseline (Stacey et al., 2000; Driver et al., 2006; Anitua et al., 2008). Despite the high heterogeneity in PRP production and application, frozen versus fresh, in almost all cases the PRP used for the treatment was autologous. Substantial variations within trials existed about eligible participants, wound etiologies, trial design, and most trials were judged to be at high risk of bias due to the lack of PRP standardization, different ulcer etiology and disparity between treated and control groups. In summary, four trials treated people with ulcers of mixed etiology reporting good results in comparison to the control group (Driver et al., 2006; Li et al., 2012; Moraes et al., 2014), three trials treated people with venous leg ulcers (Senet, 1990; Stacey et al., 2000; Mj et al., 2016) and three people with diabetes and foot ulcers (Driver et al., 2006; Kakagia et al., 2007; Mj et al., 2016). Nine out of 10 studies compared PRP plus standard care with standard care alone (with or without placebo). One study in people with diabetes evaluated PRP in the context of protease-modulating matrix (Kakagia et al., 2007). Another meta-analysis on clinical trials with a total of 477 diabetic patients compared standard care plus autologous or allogeneic PRP treatment versus standard care and showed consistent, reliable, significant beneficial effect of PRP administration on DFU healing, with no differences in wound complications or recurrences, and a decrease in the risk of adverse events in PRP-treated ulcers (del Pino-Sedeño et al., 2019).

PRP prepared from the blood of a single patient (autologous PRP) can present a significant variability in platelet and other blood component concentrations Moreover, patients with specific health status (diabetics, immune-compromised, and hypomobile) or individuals with age-related problems (neonatal, infant, elderly people) are not retained ideal candidates for the treatment with autologous platelet derivatives (Anitua et al., 2008; Backly et al., 2011; Greppi et al., 2011; Aatonen et al., 2012; Andia and Maffulli, 2013; Ulivi et al., 2014; Picard et al., 2015). A standardized PRP production from pools of human certified buffy coats (allogeneic PRP) has been proposed to reduce PRP variability and to obtain an “off the shelf” product with a well-defined platelet and growth factor concentration (Muraglia et al., 2014, 2015). Indeed, an allogeneic product obtained from a pool of healthy individuals could represent an efficient alternative to the use of autologous PRP. Allogeneic PRP can be stored as blood bank of platelet concentrate with defined cell count and growth factor amount, from donors tested for history of infectious diseases and homologous for ABO and Rh antigens (H-PRP) (Jeong et al., 2010).

An allogeneic PRP or platelet-derived product could reduce the risk of poor standardization. However, the possibility of an immune reaction should be considered. Bottegoni et al. (2016) first published results of a large-scale trial aimed at evaluating the safety and efficacy of allogeneic human PRP on patients suffering from moderate osteoarthritis. Reiterated PRP injections for 2 months showed a statistically significant improvement in all patients within the treatment period. Results worsened during the 4 months of follow-up, particularly in patients older than 80 years, confirming published results reporting PRP decreased efficacy with increasing age (Andia and Maffulli, 2013). Most importantly, this study highlights non-side effects apart from a transitory intra-articular burning. These findings are in line with those obtained by Jang et al. on homologous allogeneic platelet gel treatment of recalcitrant skin ulcers in hypomobile patients (Greppi et al., 2011). Also in this case, no adverse reactions were observed with results that are comparable, in terms of healing, to those obtained with autologous treatment. Analogous results were described by Shan et al. (2013) on DFU treatment with PRP, with an enhancement and acceleration of lower extremity wound healing.

More recently, two articles investigated the effects exerted by an allogeneic non-homologous PRP. In these studies, performed in a mouse model of diabetes, the xenogeneic platelet-rich gel enhanced the healing process without adverse reaction (Spanò et al., 2017). These observations agree with previously reported data where, for the first time, researchers evaluated the immunogenicity of allogeneic non-homologous PRP injection in rabbit large bone defects (Zhang et al., 2013). In this case, no inflammatory effects were observed, but only a slight increase in the amount of peripheral CD4+ T cells. A possible explanation for these findings could be found in the ability of the allogeneic PRP to modify the wound microenvironment. To this regard, we published that allogeneic PRP affects human monocyte differentiation to immature dendritic cells, triggering the generation of a different immune-modulatory subset, able to induce an anti-inflammatory milieu (Papait et al., 2018). We also compared the leukoreduction effect of the PRP on monocyte differentiation, and not significant differences were observed in comparison to standard PRP products.

The aforementioned meta-analysis on selected clinical trials evaluating PRP for DFU treatment (del Pino-Sedeño et al., 2019) included three studies with allogeneic PRP. Authors did not specifically compared efficacy of autologous vs. allogeneic preparations. However, outcomes of the different studies were very consistent in all parameters analyzed, suggesting a similar beneficial effect of the two preparations. A recently published clinical trial evaluating the effect of allogeneic PRP as compared to autologous PRP reported a similar improvement of both treatments with respect to control group, although a better healing rate trend was observed in ulcers treated with the allogeneic preparations. Adverse reactions were not reported for both treatments. These data confirm that allogeneic PRP could be used as a beneficial cell therapy supplement when autologous PRP is not available or difficult to obtain (He et al., 2020).

Clinical evidence, including ours, confirms that the treatment with allogeneic platelet derivatives allows healing also of ulcers hard to treat and slow to heal, which would not benefit of a “standard traditional” treatment. If the efficacy of quality controlled allogeneic PRP is confirmed by additional studies, with this relatively low-cost treatment no longer would patients need to undergo more sophisticated invasive procedures. This would be particularly important for patients who are severely disabled (i.e. bed sores, type 2 diabetes ulcers). Prompt and effective long-term treatment of ulcers and chronic wounds will reduce disease complications (i.e. infections, amputation) and mortality rate.

## 4 Discussion and Conclusion

Cutaneous chronic wounds, and in particular chronic ulcers in diabetes, represent a major clinical and societal problem in continuous growth because of population aging. Different treatments have been proposed: biological, surgical, and physical. However, most of these treatments are palliative and none of them can be considered fully satisfactory. Cell-based therapies, including stem cell conditioned medium and cell released microvesicles, together with platelet-rich plasma (PRP) treatments, are today innovative therapeutic approaches that share as common goal the reactivation of silent endogenous regeneration processes. In most cases, cells and PRP are obtained from patients as autologous preparations.

Positive preclinical data suggested the adoption of cell therapy on diabetic patients to promote healing of chronic ulcers unresponsive to standard care. Since preclinical data were mainly obtained in rodent models, that only partly recapitulate features of human diabetic wounds, and because preclinical studies often suffer from non-rigorous setting and analysis, so far, the translation to the clinic was often disappointing. Well-structured, large, highly controlled clinical trials are therefore needed to test cell therapy efficacy in diabetic ulcers.

*Ex vivo* expanded Mesenchymal Stem Cells from different body sites, either of the patient or of a donor individual, manifest high potentialities in promoting healing of diabetic wounds as compared to skin grafts. However, despite the positive results already obtained, due to rules imposed by the regulatory agencies (United States Food and Drug Administration, European Medicines Agency, National Agencies), the need of highly sophisticated cell culture facilities, and the consequent high cost of the procedures, the MSC therapeutic approach cannot be easily applied to patients of largely diffuse pathologies, such as chronic diabetic ulcers. Moreover, for autologous MSCs, the logistics to obtain a biopsy from the patient, expanding the cells in the culture lab, and returning to the patient the final product as a preparation to be immediately used, makes the approach even more difficult to be adopted. It is also to note that in metabolic disorders, such as diabetes or in elderly individuals, cell number reduction and defective paracrine activity could hamper the effectiveness of autologous cell-based therapy.

As for allogeneic therapy, a safety issue related to allo-recognition and rejection may arise as a problem. Preliminary evidence on MSC therapy indicates no major problems following allogeneic cell topical administration. However, a careful evaluation is needed before translation of allogeneic cells to the clinical practice. It is now widely recognized that the therapeutic activity go to head of the transplanted cells strongly relies on their secretome and on released microvesicles. Indeed, secretome and released microvesicles hold great promise as a therapeutic option in regenerative medicine. However, the same regulatory restrictions applying to pharmaceuticals apply to allogeneic stem cells, cell secretome or cell-released microvesicles.

Great promises rely on the use of PRP that could be easily prepared with low invasive techniques and at a relatively low cost. Moreover, at variance with cell and cell-derived products, in most countries PRP is not classified as a pharmaceutical, and it is considered a blood transfusion product. A critical issue with PRP therapy is the lack of standardization in the preparation procedures. This problem is at the root of the discrepancies in results reported in literature. Most of reported PRP treatments have utilized a product obtained from the blood patients (autologous PRP). An allogeneic PRP obtained from a pool of healthy subjects could be a better alternative to the use of autologous PRP. Allogeneic PRP preparations can be quality controlled before their use and can be stored in a freeze-dried form as “off the shelf” product for a future use.

It is expected that allogeneic PRP will have the same, if not better, performance, than autologous PRP. The few published reports seem to confirm this expectation. However, large randomized clinical trials comparing allogeneic PRP to other treatments are still missing. This is mandatory for a reliable, efficient application on a large scale of allogeneic PRP in the clinical practice.
